# Advancing Transportation Equity and Safety Through Autonomous Vehicles

**DOI:** 10.1089/heq.2023.0107

**Published:** 2024-03-07

**Authors:** Johnathon P. Ehsani, Jeffrey P. Michael, Takeru Igusa, Joshua Mueller, Chia-Hsiu Chang, Gayane Yenokyan

**Affiliations:** ^1^ Johns Hopkins Bloomberg School of Public Health, Baltimore, Maryland, USA.; ^2^ Johns Hopkins Whiting School of Engineering, Baltimore, Maryland, USA.; ^3^ Johns Hopkins Applied Physics Laboratory, Laurel, Maryland, USA.

**Keywords:** autonomous vehicles, public support, social benefit

## Abstract

Motor vehicle crashes are a leading cause of death in the United States, and disproportionately impact communities of color. Replacing human control with automated vehicles (AVs) holds the potential to reduce crashes and save lives. The benefits of AVs, including automated shuttles, buses, or cars could extend beyond safety to include improvements in congestion, reductions in emissions, and increased access to mobility, particularly for vulnerable populations. However, AVs have not attained the level of public trust that has been expected, given their potential to save lives and increase access to mobility. Public opinion surveys have highlighted safety and security concerns as reasons for this lack of confidence. In this study, we present the findings of an experiment we conducted to actively shift mindsets on AVs toward advancing health equity. We demonstrate through a nationally representative sample of 2265 U.S. adults that the public support for AVs can be improved by expanding their scope of application to include advancing social benefit. The survey began with questions on respondent's support for AVs based on *a priori* knowledge and beliefs. Consistent with prior surveys, baseline support (strong support and some degree of support) was low at 26.4% (95% confidence interval 24.0–29.0). After introducing information about how AVs could be used to provide mobility for older adults, those with limited income, or the vision-impaired, respondents were asked to reassess their support for AVs. Support significantly increased to include the majority of respondents. By prioritizing the deployment of AVs to serve individuals and communities in greatest need of mobility, AVs would not only demonstrate compelling social value by reducing disparities but would also gain widespread public support among the U.S. public.

## Introduction

Motor vehicle crash deaths are increasing at an historic rate in the United States, with an estimated 42,915 people dying in crashes on U.S. roads in 2021.^1^ The population-based crash death rate in the United States (11.1 per 100,000 population) is the highest across all high-income countries.^2^ Traffic deaths are not equally distributed across the population, but disproportionately affect communities of color and low-income neighborhoods.^3^ Crash deaths rates are twice as high among American Indian/Alaska Native children and adults than for the general population, and Black people of all ages are close to 20% more likely to die in a crash than the national average.^4^

Replacing human control with fully automated vehicles (AVs) that do not require a human driver holds the potential to reduce crashes and save lives. The benefits of automated cars, shuttles, or buses could extend beyond safety to include improvements in congestion, reductions in emissions, and increased access to mobility, particularly for vulnerable populations.^5^ However, billions of dollars in technology investment and a decade of public education have failed to assuage public concerns about AV safety. Annual surveys indicate that fewer than one-third of Americans trust AVs to reduce the likelihood of a crash relative to human drivers.^6^ Anxieties about AVs range from software and hardware failures to moral questions about automated decisions that could affect who is injured in a crash.^7^

Safety advocates argue that it is essential to secure public trust and support for AVs.^6^ Part of the challenge is that conventional approaches may not be suitable for assessing their risks.^8^ For example, the idea that AVs could be tested on public roadways until they demonstrate a specific benchmark of safety that has been challenged by an analysis suggesting that hundreds of millions or billions of miles of testing would be needed, potentially taking decades.^8^ In the absence of standard benchmarks, support for the adoption of AVs may need to come from other dimensions of value.

In a prior work, we argue that one approach to increasing acceptance is to expand public perception of AV value beyond safety to include their potential effect on wider societal benefits.^9,10^ Such a strategy could not only increase the perceived value of AVs but also make public perception more resilient to safety incidents with AVs that will inevitably occur. The following section describes an experiment we conducted to actively shift mindsets on AVs toward advancing health equity.

## Maximizing Impact

Recognizing that access to transportation is a social determinant of health and prioritizing the deployment of AVs to provide mobility to underserved populations could influence the quality of life for millions of individuals.^11^ This approach could improve public perception and accelerate the cycle of industry investment, adoption, and continued development of AVs. In a recent survey of a nationally representative sample of 2265 U.S. adults, we tested how public perceptions of AVs could be modified by an expanded conception of their societal benefit.

We fielded the Johns Hopkins Transportation Safety and Public Health Survey from May 4 to June 10, 2022, using NORC's AmeriSpeak Panel. AmeriSpeak is a probability-based panel designed to be representative of the U.S. adult population. The panel is sourced from NORC's area probability sample and from a U.S. Postal Service address-based sample covering 97% of U.S. households.^12^ The sample for the Johns Hopkins survey was drawn from this panel and administered via telephone and online, in English and Spanish. NORC obtains informed consent before enrolling individuals in the panel. The Johns Hopkins Bloomberg School of Public Health Institutional Review Board deemed this study not human participant research. Panel members were compensated $4 for their participation in the survey.

Respondents were initially asked a series of questions about their support for AVs that benchmarked their responses against prior national surveys. We then measured the prevalence of support for a range of AV deployment scenarios in the overall sample using a five-point scale. Questions were sequenced to measure initial baseline views and how these changed in response to different deployment scenarios. Respondents were first asked a number of items related to trust and whether they “would support or oppose fully automated self-driving cars on the streets in your community.”

Respondents were then asked to read the following statement and answer three subsequent questions: “Because they don't have a driver, fully automated self-driving cars could make the cost of a trip very affordable. This could allow free or very cheap rides to be provided for people who now have trouble getting around, such as older adults who cannot drive, people from lower income households who don't have reliable access to transportation, and people who are vision impaired.” Following this statement, respondents were asked, “if fully automated self-driving cars were widely used to provide transportation to older adults who previously had difficulty getting around, would you support or oppose fully automated self-driving cars on the streets in your community?” Similar items were asked about individuals with lower incomes and with vision impairments.

The survey was programmed so that respondents could not go back and change their answers to the initial question after being presented with the subsequent statement. A variable for “overall support” and “overall oppose” was created by combining “strongly” and “somewhat” responses for both support and opposition. Analyses incorporated sampling weights to generate nationally representative estimates. The survey completion rate was 31.6%.

## Broadening Support

**FIG. 1.**
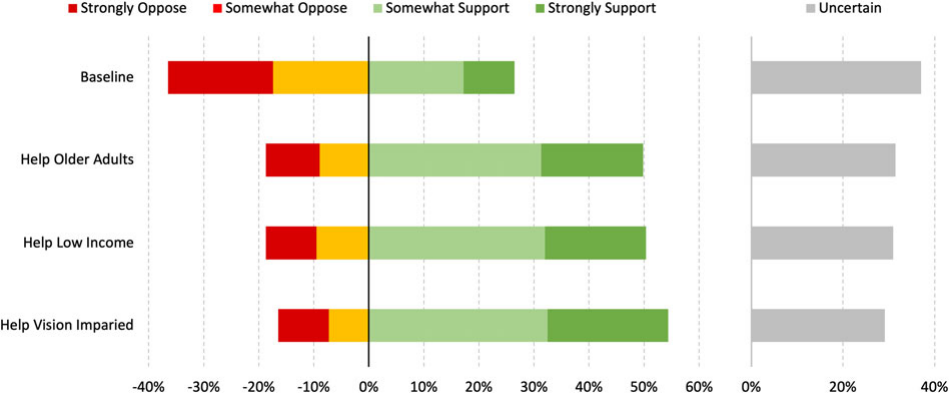
Baseline support for AVs and shift in support for AVs based on deployment scenarios. AVs, automated vehicles.

While initial enthusiasm for AVs was low with 26.4% [95% confidence interval (CI): 24.0–29.0]) of adults either strongly or somewhat supporting them, overall support significantly increased to 49.7% [95% CI: 46.7–52.7], following the description of the scenario of using AVs for older adults. A similar magnitude of change in overall support was seen for providing transportation for people of lower income who previously had trouble getting around, increasing to 50.5% [95% CI: 47.4–53.5]. The largest increase in support was observed for the scenario providing transportation for the vision impaired, increasing to 54.3% [95% CI: 51.3–57.3]. All increases in support relative to the baseline were statistically significant (Fig. 1).

To benchmark the sample in this study to prior surveys, we administered questions fielded in previous national surveys to measure support for AVs before we presented them with the scenarios described above. In a 2018 survey of U.S. adults, 50% of respondents of a Deloitte survey felt that AVs will not be safe when they are introduced.^13^

In this sample, the percentage that reported that AVs will not be safe was 48.3%. In a national survey of U.S. adults conducted by Gallup, respondents were asked, “Once fully automated self-driving cars are certified by government auto safety regulators, do you think you would want to use one?” In the Gallup survey, 38% of respondents said they would wait a while to use an AV, and 52% said they would never use one.^14^ In this study, 48.0% [95% CI: 45.0–51.0] said they would wait a while and 34.6% [95% CI: 31.8–37.6] said they would never use one. These findings suggest that before receiving the prompt about the social benefit of AV, the respondents in this sample held ambivalent views toward AVs that were comparable to those shown in prior work.

In these analyses, we excluded individuals who either had missing data for the baseline support for AVs (*n*=8) or had missing responses on support for AVs for any social purpose (*n*=51). Those who were initially strongly supportive of AVs (*n*=211) were removed from the analysis measuring a shift in perception, because they were not eligible to become any more supportive in response to the scenarios.

## Public Policy Opportunity

The potential of considerably enhancing public support for AVs by pursuing deployment scenarios that prioritize the needs of individuals who are in greatest need of mobility has implications for practitioners, policymakers, and the industry. Creative solutions that maximize the utility of AVs could include ancillary support services to help older adults or individuals with vision impairments with door-to-door and/or door-through-door assistance, and with seat belts or packages when they are utilizing AVs. State and city transportation regulators could require that mobility providers dedicate a certain proportion of AV trips to serve these populations. Currently, few jurisdictions have these requirements in place.^15^ Such efforts would have the dual purpose of serving to increase the perceived value of AVs and addressing existing mobility needs. This process could begin with community engagement and an examination of the physical infrastructure of underserved neighborhoods to ensure that AVs are adequately tested in these environments. A series of large-scale demonstration of projects throughout the United States, such as efforts in Trenton, NJ^16^ could advance efforts to facilitate public trust and acceptance of AVs. The industry could further advance public trust by investing in public projects demonstrating the social benefit of AVs that would complement those required by government regulators. AVs offer a range of potential social benefits, promising to substantially reduce traffic deaths which remain a leading cause of death worldwide and increase mobility among underserved populations. To realize these benefits, AVs need to be widely accepted and deployed as a public good rather than as simply another transportation mode for those who can afford them. Prioritizing the deployment of AVs to serve the needs of individuals and communities that are in the greatest need of mobility could simultaneously increase public support of the technology and demonstrate compelling social value by reducing disparities and providing access to the social determinants of health. In addition, practitioners should also work to understand and think creatively about how to address the specific needs of vulnerable populations in future AV demonstration projects or interventions.

## Abbreviations Used

AV: automated vehicle
CI: confidence interval
